# Discordance in HER2 Status in Gastro-esophageal Adenocarcinomas: A Systematic Review and Meta-analysis

**DOI:** 10.1038/s41598-017-03304-9

**Published:** 2017-06-09

**Authors:** A. Creemers, E. ter Veer, L. de Waal, P. Lodder, G. K. J. Hooijer, N. C. T. van Grieken, M. F. Bijlsma, S. L. Meijer, M. G. H. van Oijen, H. W. M. van Laarhoven

**Affiliations:** 1 Cancer Center Amsterdam, Laboratory for Experimental Oncology and Radiobiology, Center for Experimental and Molecular Medicine, AMC, Meibergdreef 9, 1105 AZ Amsterdam, The Netherlands; 2 Cancer Center Amsterdam, Department of Medical Oncology, AMC, Meibergdreef 9, 1105 AZ Amsterdam, The Netherlands; 3 Department of Methodology and Statistics/Department of Medical and Clinical Psychology, Tilburg University, Warandelaan 2, 5037 AB Tilburg, The Netherlands; 4 Department of Pathology, AMC, Meibergdreef 9, 1105 AZ Amsterdam, The Netherlands; 5 Department of Pathology, VUMC, De Boelenlaan 1117, 1081 HV Amsterdam, The Netherlands

## Abstract

Trastuzumab combined with chemotherapy is standard of care for HER2 positive advanced gastro-esophageal cancers. The reported prevalence of HER2 discordance between primary tumors and corresponding metastases varies, hampering uniform patient selection for HER2 targeted therapy. This meta-analysis explores the influence of HER2 assessment methods on this discordance and investigates the prevalence of HER2 discordance in gastro-esophageal adenocarcinomas. PubMed, Embase and Cochrane databases were searched until January 2016. Differences in discordance rate between strict and broad(er) definitions of HER2 status were assessed using random-effect pair-wise meta-analysis. Random-effect single-arm meta-analyses were performed to assess HER2 discordance and the prevalence of positive and negative conversion. A significantly lower discordance rate in HER2 status between primary tumors and corresponding metastases was observed using a strict vs. broad definition of HER2 status (RR = 0.58, 95%CI 0.41–0.82), with a pooled discordance rate of 6.2% and 12.2%, respectively. Using the strict definition of HER2 assessment pooled overall discordance was 7% (95%CI 5–10%). The lowest discordance rates between primary tumors and corresponding metastasis are observed when using a strict method of HER2 positivity. Treatment outcomes of different studies will be better comparable if selection of eligible patients for HER2 targeted therapy is based on this strict definition.

## Introduction

Since the publication of the ToGA trial, trastuzumab with chemotherapy is standard of care for human epithelial growth factor receptor 2 (HER2) positive advanced gastro-esophageal cancer^1^. Therefore, assessment of the HER2 status is recommended for all patients with irresectable gastro-esophageal adenocarcinomas. Since the HER2 status may change in the course of disease progression and discordance in HER2 status between primary tumors and metastases has been observed, the question arises how to adequately select patients for HER2 targeted therapy.

Discordance can be observed in two ways: HER2 status may be positive in the primary tumor and negative in the corresponding metastasis – so called negative conversion – or, vice versa, negative in the primary tumor and positive in the metastasis, also known as positive conversion. Discordance can be detected in both synchronous and metachronous metastases, and both local (lymph node) or distant metastases, resulting in a broad palette of intra-patient discordance in HER2 status.

Although several studies have analyzed the prevalence of HER2 status discordance, the evidence is inconsistent. In a recent review Peng and colleagues included 18 articles, focusing on gastric cancer^2^. Since then, new studies have been published, and importantly, the influence of the method of HER2 status determination has not been assessed. Discrepancy in the definition of HER2 positivity and the method of assessment between studies may at least partly explain the observed inconsistency.

Currently, the consensus method of HER2 status assessment entails immunohistochemistry (IHC), scored using the system by Hofmann *et al*.^3^. Even so, there are several studies reporting on HER2 status not compliant to this consensus scoring system, which were included in the meta-analyses of Peng and colleagues^2^. Furthermore, even if studies use the Hofmann scoring system, frequently not only IHC 3+ scoring tumors but also IHC 2+ tumors are reported to be HER2 positive without further *in situ* hybridization (ISH) testing. Considering the selection of eligible patients for trastuzumab treatment, it is important to notice that the ToGA trial included patients with either HER2 IHC 3+ scores and/or amplification of the HER2/neu gene by fluorescence *in-situ* hybridization (FISH). However, subgroup analysis showed no benefit of the addition of trastuzumab to chemotherapy for patients with FISH positive, IHC 0/1+ tumors. Hence, in daily practice the ASCO HER2 guideline, published in November 2016, is usually followed^4^: Tumor specimens with strong staining of more than 10% of the tumor cells, IHC 3+, are defined HER2 positive. In addition, all specimens scoring IHC 2+ are further investigated by means of *in situ* hybridization. Those IHC 2+ tumors showing amplification of the HER2/neu gene (HER2:CEP17 ratio of 2.0, or when using a single probe >6.0 copies) are likewise confirmed HER2 positive cases and patients may be treated with trastuzumab.

For the purpose of this review, we used strict and broad(er) definitions of HER2 assessment. The strict definition of HER2 detection consists of HER2 IHC scoring according to the consensus scoring system of Hofmann *et al*.^3^ with HER2 positivity defined as IHC score 3+ or IHC score 2+ with an amplification of the *HER2/neu* gene by *in situ* hybridization (ISH). The broad definitions include IHC scores 2+ and 3+ marked HER2 positive (irrespective of ISH) or amplification of the *HER2/neu* gene without IHC analysis. This review explores the influence of these different definitions of HER2 assessment on the HER2 status discordance rate between primary tumors and corresponding metastasis in both gastric and esophageal adenocarcinomas. Furthermore, it provides an updated systematic review and meta-analysis of the HER2 discordance rate in gastro-esophageal cancer.

## Results

### Study characteristics

**Figure 1:**
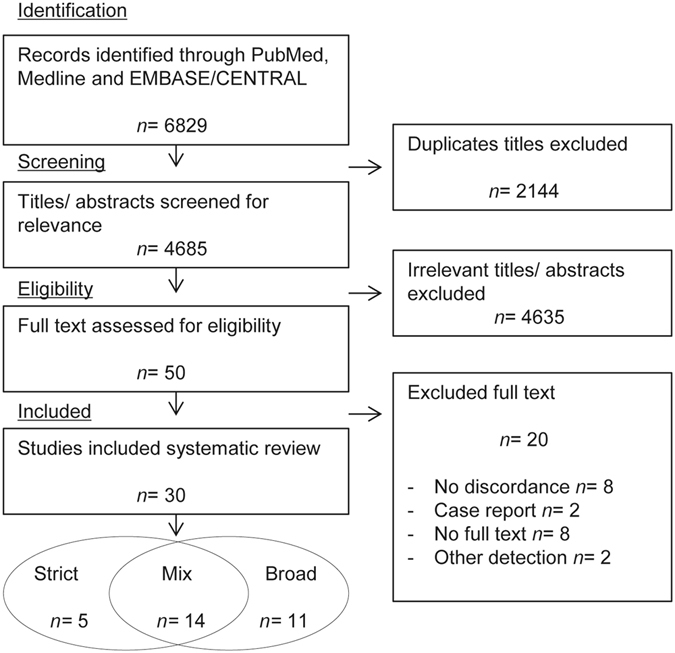
Flow chart of eligible studies for meta-analysis. Strict definition of HER2 assessment: HER2 positivity defined as IHC score 3+ or IHC score 2+ with an amplification of the HER2/neu gene by *in situ* hybridization (ISH). Broad definition: include IHC scores 2+ and 3+ marked HER2 positive (irrespective of ISH) or amplification of the HER2/neu gene without additional IHC analysis. Mix: Include data of both strict and broad definitions of HER2 status determination; articles used in pair-wise meta-analyses.

The conducted search identified 6829 articles, of which 2144 duplicate articles were removed (Fig. 1). After screening on title and abstract 50 articles were assessed as full text, of which 20 articles did not meet the selection criteria. Among these articles, eight articles did not show data on discordance, two articles did not use either the strict or a broad definition of HER2 assessment (electrophoreses and immunoblotting), two case reports were excluded and of eight articles no English text was available. Of the resulting 30 articles meeting the selection criteria, 14 contained discordance data determined using both the strict and broad definition of HER2 detection^5–18^. Five articles solely used the strict definition^19–23^ and 11 articles applied merely a broad definition of HER2 status assessment^24–34^. Quality assessment according to the adapted REMARK criteria revealed one study of low quality (Table 1)^24^. This study was excluded from all analyses. All of the other studies were of sufficient quality (Table 2). This generated discordance data comparing the strict and broad definition of HER2 detection in pair-wise meta-analyses in a population of 1207 primary tumors and their corresponding metastases^5–18^. In total 1624 cases could be included in the subsequent single arm meta-analyses of studies applying the strict definition of HER2 assessment, ranging from 29 to 250 cases per study^5–23^.

**Table 1:** Adapted REMARK criteria for Quality Assessment^39^.

Adapted REMARK criteria for Quality Assessment (1 point/criteria)
1. Case selection adequate (baselines form medical chart) (=1 point)
2. Case selection representative (=1 point)
3. Received (neo)adjuvant therapy before tumor sampling: yes/no/NR (=1 point if not NR)
4. Reporting at least the following specimen characteristics: location of primary tumor (esophageal, GEJ, stomach), location of metastasis (local/distant), timing of metastasis (metachronous/synchronous), type of sampling (biopsy/resection specimen) (=1 point)
5. Clear description of the method of HER2 status determination: IHC/ISH and used scoring systems (=1 point)
6. A clear description of the flow of patients through the study (=1 point)
7. A clear description of the reasons of dropout (=1 point)

Studies are appointed one point for each item, half a point is allocated in case of ambiguity.

**Table 2:** Quality assessment according to adapted REMARK criteria.

Study	C1	C2	C3	C4	C5	C6	C7	Total
Bozzetti^10^	+/−	+	+	+	+	+	+	6.5
Chariyalertsak^24^	+/−	+	−	+	+	−	+	4.5
Cho^9^	+	+	+	+	+	+/−	+	6.5
Fassan^18^	+	+	+	+	+	+	+	7
Fusco^12^	+	+	−	+	+	+	+/−	5.5
Geng^25^	+/−	+	+	+	+	+	+	6.5
Gumusay^17^	+	+	+/−	+	+	+	+	6.5
Hedner^19^	+	+	+	+	+	+	+	7
Ieni^16^	+	+	+	+	+	+	+	7
Kim^40^	+	+	+	+	+	+	+	7
Kim^6^	+	+	−	+	+	+	+	6
Kochi^13^	+/−	+	−	+	+	+	+	5.5
Konig^26^	+	+	+	+	+	+	+	7
Marx^27^	+	+	−	+	+	+	+	6
Ougolkov^28^	+	+	+	+	+	+	+	7
Pagni^11^	+/−	−	+	+	+	+	+	5.5
Park^21^	+	+	+	+	+	+	+	7
Qiu^29^	+/−	+	+	+	+	+	+	6.5
Reichelt^30^	+	+	+	+	+	+	+	7
Saito^31^	+/−	+	−	+	+	+	+	5.5
Schoppmann^22^	+	+	+	+	+	+	+	6.5
Schoppman^23^	+	+	+	+	+	+	+	7
Selcukbirick^34^	+	+	+	+	+	+	+	7
Shibata^15^	+/−	+	+	+	+	+	+	6.5
Tsapralis^8^	+	−	+/−	+	+	+	+	5.5
Walch^32^	+	+	+	+	+	+	+	7
Wei^5^	+/−	+	+	+	+	+	+	6.5
Wei^16^	+	+	+	+	+	+	+	7
Wong^14^	+/−	+	−	+	+	+	+	5.5
Yu^33^	+	+	−	+	+	+	+	6

Studies are appointed one point for each item, half a point is allocated in case of ambiguity. Maximum score of 7 points.

The main characteristics of the included articles are shown in Table 3. The articles were published between June 1994 and January 2016. Twelve studies applied both IHC and ISH to assess the HER2 status, five used ISH and the remaining determined HER2 status discordance by means of IHC. Seventeen articles included cases with the primary tumor located in the stomach or GEJ, six studies contained data of cases with the primary tumor solely in the esophagus and one study showed data of tumors located either in the esophagus, GEJ or stomach. The majority of studies analyzed adenocarcinomas. Squamous cell carcinomas were analyzed in four articles; all of these cases were analyzed separately from the adenocarcinoma group. Most of the studies (n = 17) focused on discordant HER2 status of synchronous locoregional lymph nodes in resection specimen. Six studies included distant metastases only, five studies showed data of both locoregional lymph node and distant metastases. The two remaining studies examined locoregional recurrences. Only two articles explicitly stated that they included patients who had received (neo)adjuvant treatment.

**Table 3:** Study characteristics of studies included in meta-analysis.

Study	Method	No. of subjects	Group	Location	Histology	Metastases	Timing	Sampling	Treatment	Ethnicity	Design
Bozzetti^10^	IHC	39	Mix	GEJ Stomach	Adenocarcinoma	Distant	Synchronous Metachronous	Surgery Biopsy	None	Western	Retrospective
Chariyalertsak^24^	IHC-other	97	Broad	GEJ Stomach	Adenocarcinoma	Locoregional lymph node	Synchronous Metachronous	Surgery Biopsy	NR	Asian	Retrospective
Cho^9^	IHC	41	Mix	GEJ Stomach	Adenocarcinoma	Distant	Synchronous Metachronous	Surgery	None	Asian	Retrospective
Fassan^18^	IHC	47	Mix	GEJ Stomach	Adenocarcinoma	Locoregional lymph node	Synchronous	Surgery	None	Western	Retrospective
Fusco^12^	IHC	154	Mix	GEJ Stomach	Adenocarcinoma	Locoregional lymph node	Synchronous	Surgery	NR	Western	Retrospective
Geng^25^	IHC2–3	110	Broad	GEJ Stomach	Adenocarcinoma	Locoregional lymph node	Synchronous	Surgery	None	Asian	Retrospective
Gumusay^17^	IHC SISH	74	Mix	GEJ Stomach	Adenocarcinoma	Distant	Synchronous Metachronous	Surgery Biopsy	NR	Asian	NR
Hedner^19^	IHC SISH	70	Strict	Esophagus GEJ Stomach	Adenocarcinoma	Locoregional lymph node	Synchronous	Surgery	None	Western	Retrospective
Ieni^16^	IHC FISH	108	Mix	GEJ Stomach	Adenocarcinoma	Locoregional lymph node	Synchronous	Surgery	None	Western	Retrospective
Kim^40^	IHC	222	Strict	GEJ Stomach	Adenocarcinoma	Locoregional lymph node	Synchronous	Surgery	None	Asian	Retrospective
Kim^6^	IHC FISH	250	Mix	GEJ Stomach	Adenocarcinoma	Locoregional lymph node Distant	Synchronous Metachronous	Surgery Biopsy	NR	Asian	Retrospective
Kochi^13^	IHC FISH	102	Mix	GEJ Stomach	Adenocarcinoma	Locoregional lymph node	Synchronous	Surgery	NR	Asian	Retrospective
Konig^26^	FISH	158	Broad	Esophagus	Adenocarcinoma Squamous cell carcinoma*	Locoregional lymph node	Synchronous	Surgery	None	Western	Retrospective
Marx^27^	FISH	49	Broad	GEJ Stomach	Adenocarcinoma	Locoregional lymph node	Synchronous	Surgery	NR	Western	Retrospective
Ougolkov^28^	IHC-other	16	Broad	GEJ Stomach	Adenocarcinoma	Distant	Synchronous	Surgery Biopsy	None	Asian	Retrospective
Pagni^11^	IHC FISH	34	Mix	GEJ Stomach	Adenocarcinoma	Locoregional lymph node	Synchronous	Surgery Biopsy	None	Western	Retrospective
Park^21^	IHC FISH	175	Strict	GEJ Stomach	Adenocarcinoma	Locoregional recurrence Distant	Synchronous Metachronous	Surgery Biopsy	Some	Asian	Retrospective
Qiu^29^	IHC2–3	99	Broad	GEJ Stomach	Adenocarcinoma	Locoregional lymph node	Synchronous	Surgery	None	Asian	Prospective
Reichelt^30^	FISH	114	Broad	Esophagus	Adenocarcinoma Squamous cell carcinoma*	Locoregional lymph node Distant	Synchronous	Surgery	None	Western	Retrospective
Saito^31^	IHC2–3	91	Broad	GEJ Stomach	Adenocarcinoma	Distant	Synchronous Metachronous	Surgery Biopsy	NR	Asian	Retrospective
Schoppmann^22^	IHC CISH	58	Strict	Esophagus	Adenocarcinoma Squamous cell carcinoma*	Locoregional recurrence	Metachronous	Surgery Biopsy	All	Western	Retrospective
Schoppman^23^	IHC CISH	205	Strict	Esophagus	Adenocarcinoma Squamous cell carcinoma*	Locoregional lymph node Distant	Synchronous Metachronous	Surgery	None	Western	Prospective
Selcukbirick^34^	SISH	74	Broad	GEJ Stomach	Adenocarcinoma	Locoregional lymph node	Synchronous	Surgery	None	Western	Retrospective
Shibata^15^	IHC FISH	37	Mix	GEJ Stomach	Adenocarcinoma	Distant	Synchronous Metachronous	Surgery Biopsy	None	Asian	Retrospective
Tsapralis^8^	IHC CISH	45	Mix	GEJ Stomach	Adenocarcinoma	Locoregional lymph node	Synchronous	Surgery	NR	Western	Retrospective
Walch^32^	FISH	5	Broad	Esophagus	Adenocarcinoma	Locoregional lymph node	Synchronous	Surgery	None	Western	Retrospective
Wei^5^	IHC	39	Mix	Esophagus	Squamous cell carcinoma	Locoregional lymph node	Synchronous	Surgery	None	Asian	Retrospective
Wei^7^	IHC	29	Mix	GEJ Stomach	Adenocarcinoma	Locoregional lymph node Distant	Synchronous	Surgery	None	Asian	Retrospective
Wong^14^	IHC SISH	43	Mix	GEJ Stomach	Adenocarcinoma	Locoregional lymph node Distant	NR	Surgery Biopsy	NR	Western	Retrospective
Yu^33^	IHC-other	262	Broad	GEJ Stomach	Adenocarcinoma	Locoregional lymph node	Synchronous	Surgery	NR	Asian	Retrospective

GEJ: Gastro-esophageal junction tumors. *: Separate analysis. Abbreviations: CISH: Chromogenic in situ hybridization. FISH: Fluorescence in situ hybridization. SISH: Silver in situ hybridization. IHC: immunohistochemistry. NR: not reported. Strict: include data of HER2 positivity defined as IHC score 3+ or IHC score 2+ with an amplification of the HER2/neu gene by in situ hybridization (ISH). Broad: include IHC scores 2+ and 3+ marked HER2 positive (irrespective of ISH) or amplification of the HER2/neu gene without additional IHC analysis. Mix: Include data of both strict and broad definitions of HER2 status determination; articles used in pair-wise meta-analyses.

### Meta-analyses of strict vs. broad definition of HER2 detection

**Figure 2:**
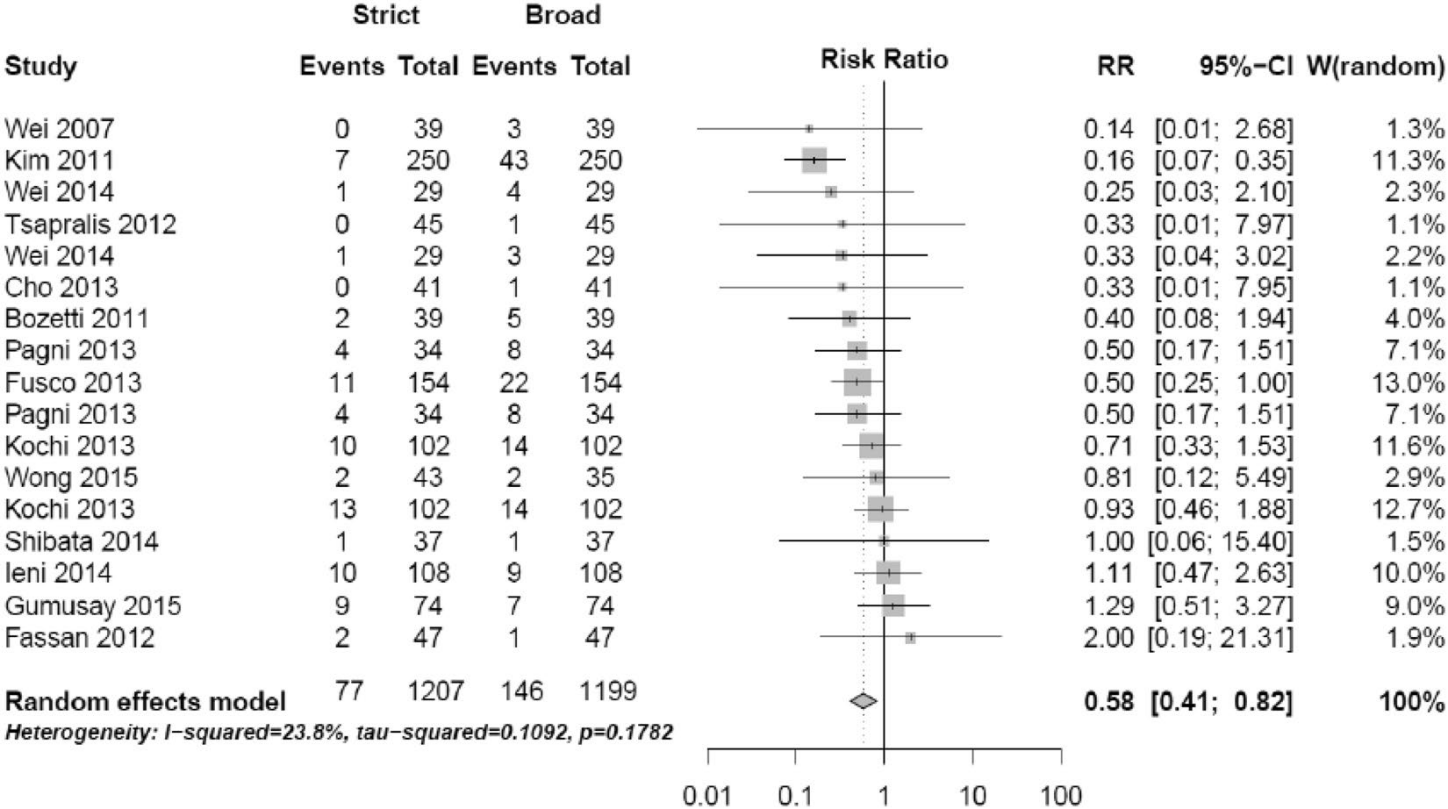
Random-effect pair-wise meta-analysis of total HER2 status discordance of strict vs. broad methods of HER2 detection.

Among the articles meeting the selection criteria 14 articles could be included in random-effect pair-wise meta-analyses, resulting in a total of 1207 primary tumors and corresponding metastases (Fig. 2). A significantly lower discordance rate in HER2 status between primary tumors and metastases was observed using the strict versus the broad definition of HER2 status (RR = 0.58, 95%CI 0.41–0.82), with a pooled discordance rate of 6.2% (range 0– 12.7%) and 12.2% (range 2.1– 23.5%), respectively. No statistically significant heterogeneity was detected (I^2^ = 23.8%, p = 0.18). The effect of the method of HER2 determination on HER2 status discordance was more pronounced in regional metastasis than in distant metastasis (RR = 0.68, 95%CI 0.49–0.94) vs. (RR = 0.81, 95%CI 0.40–1.65). However, the test for subgroup differences was not significant (p = 0.65, I² = 0%).

**Figure 3:**
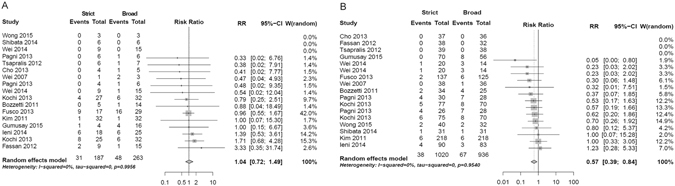
Random-effect pair-wise meta-analyses of (**A**). Positive to negative HER2 status discordance (negative conversion) of strict vs. broad methods of HER2 detection (**B**). Negative to positive HER2 status discordance (positive conversion) of strict vs. broad methods of HER2 detection.

Random-effect pair-wise meta-analyses of positive to negative HER2 status conversion and vice versa using strict vs. broad definitions of HER2 assessment display significantly more negative to positive HER2 status discordance if broad definitions of HER2 status determination are applied, RR = 1.04, 95%CI (0.72–1.49) vs. RR = 0.57 95%CI (0.39–0.84), respectively (Fig. 3). Both analyses demonstrated no significant heterogeneity (I² = 0%, p = 0.99 and I² = 0%, p = 0.94).

### HER2 status discordance between primary tumor and corresponding metastases

**Figure 4:**
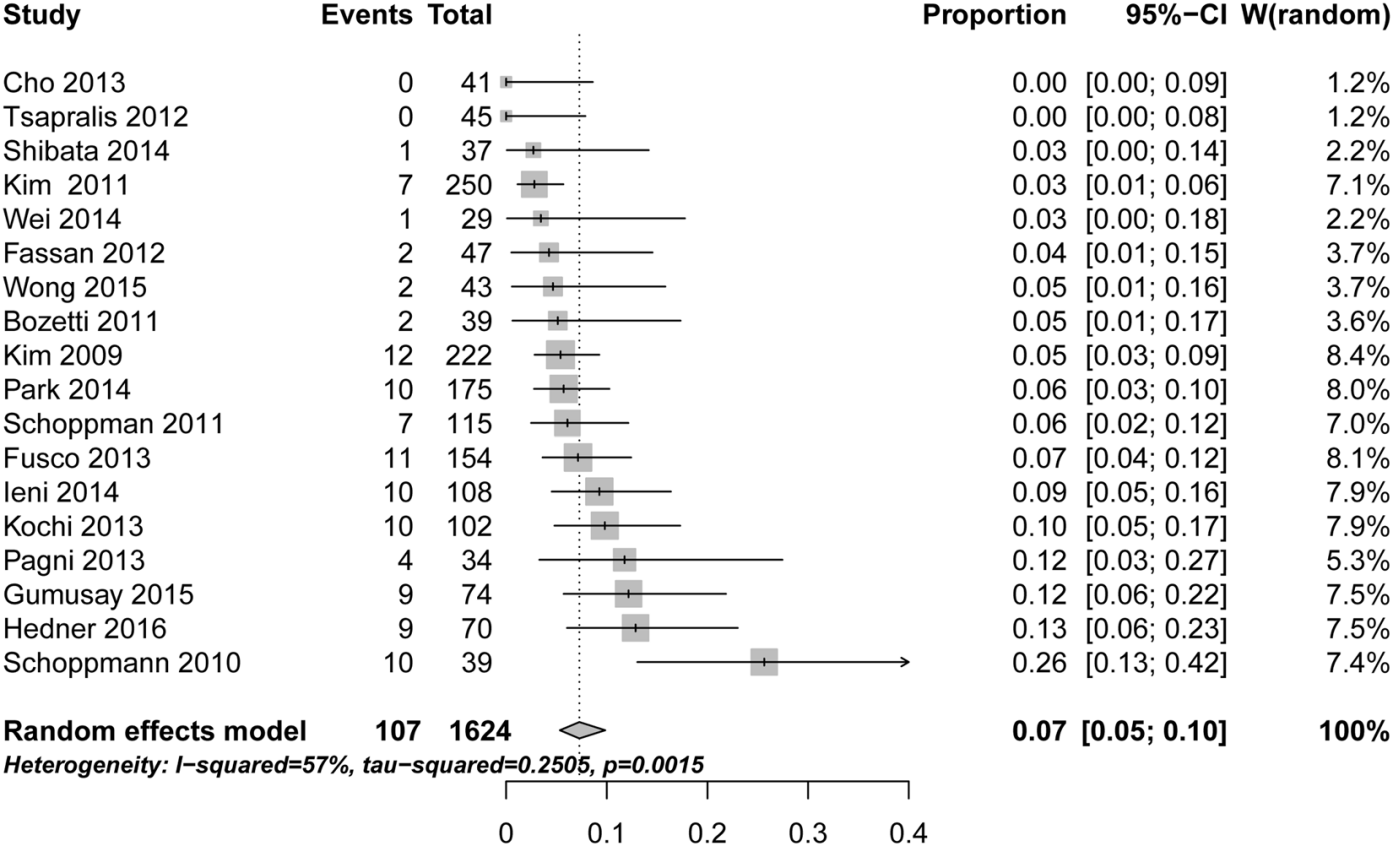
Random-effect single-arm meta-analysis of proportions of discordant HER2 status in gastro-esophageal adenocarcinomas.

Random-effect single-arm meta-analyses were performed on 18 articles using the strict definition of HER2 status assessment with data on proportion of discordance in gastro-esophageal adenocarcinomas. Discordance was detected in 107 of the 1624 cases, pooled overall HER2 status discordance between primary tumors and corresponding metastases was 7%, 95%CI (5–10%) ranging from 0% to 26% (Fig. 4). A significant proportion of heterogeneity was found (I² = 57%, p = 0.0015).

**Figure 5:**
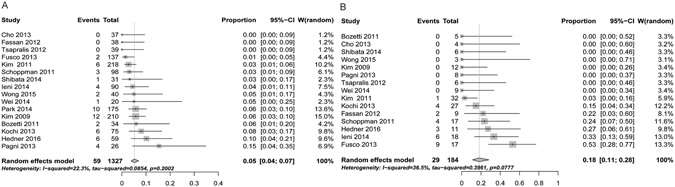
Random-effect single-arm meta-analysis of (**A**). Proportions of positive to negative discordant HER2 status in gastro-esophageal adenocarcinomas (**B**). Proportions of negative to positive discordant HER2 status in gastro-esophageal adenocarcinomas.

Evaluating the proportion of positive to negative conversion and negative to positive conversion, more positive to negative conversion was observed, 18% (95%CI 0.11–0.28) vs. 5% (95%CI 0.04–0.07), respectively (Fig. 5). No significant heterogeneity was observed (I² = 39.5%, p = 0.07 and I² = 22.3% p = 0.20).

### Sub-group analyses

The discordance rate between sub-groups showed no significant differences: method of HER2 detection (IHC3+ or IHC2+ and additional ISH), location (esophageal, GEJ/stomach), type of metastasis (locoregional lymph node or distant), timing of metastasis (synchronous or metachronous), sampling type (resection specimen or biopsy and resection specimen), received (neo)adjuvant therapy (yes, no or not reported), ethnicity (Asian or Western) and study design (retro- or prospective) (Table 4). Some of the selected studies also showed data on squamous cell carcinomas. Sub-group analysis indicated no significant difference in discordance rate between adeno- and squamous cell carcinomas.

**Table 4:** Random-effect single arm subgroup analyses of proportions of HER2 status discordance.

Type of sub-group analyses	HER2 discordance (95% CI)	Population	p-value
**Method of HER2 detection**			
IHC3+ defined HER2 positive	0.06, 95%CI (0.04–0.08)	532	0.172
IHC2+ and ISH positive defined HER2 positive	0.08, 95%CI (0.05–0.12)	1092	
**Location**			
Esophagus	0.13, 95%CI (0.03–0.44)	154	0.394
GEJ/stomach	0.07, 95%CI (0.05–0.09)	1470	
**Histology**			
Adenocarcinoma	0.07, 95%CI (0.05–0.10)	1682	0.625
Squamous cell carcinoma	0.04, 95%CI (0.01–0.13)	148	
**Type of metastasis**			
Locoregional lymph node	0.08, 95%CI (0.06–0.10)	896	0.530
Distant metastasis	0.07, 95%CI (0.04–0.11)	394	
**Timing of metastasis**			
Synchronous	0.08, 95%CI (0.06–0.10)	843	0.357
Metachronous	0.12, 95%CI (0.04–0.34)	161	
Synchronous and metachronous	0.05, 95%CI (0.03–0.10)	548	
**Type of metastasis and positive conversion**			
Locoregional lymph node	0.05, 95%CI (0.03–0.09)	767	0.961
Distant metastasis	0.05, 95%CI (0.03–0.09)	292	
**Type of metastasis and negative conversion**			
Locoregional lymph node	0.23, 95%CI (0.14–0.35)	129	0.192
Distant metastasis	0.11, 95%CI (0.03–0.29	28	
**Timing of metastasis and positive conversion**			
Synchronous	0.06, 95%CI (0.04–0.09)	755	0.216
Metachronous	0.07, 95%CI (0.04–0.13)	121	
Synchronous and metachronous	0.04, 95%CI (0.03–0.06)	586	
**Timing of metastasis and negative conversion**			
Synchronous	0.22, 95%CI (0.12–0.36)	117	0.504
Metachronous	0.25, 95%CI (0.01–0.89)	1	
Synchronous and metachronous	0.12, 95%CI (0.05–0.27)	63	
**Sampling type**			
Surgery	0.07, 95%CI (0.06–0.10)	933	0.961
Biopsy and surgery	0.05, 95%CI (0.03–0.10)	691	
**Received neo-adjuvant therapy**			
Yes	0.13, 95%CI (0.03–0.44)	214	0.715
No	0.07, 95%CI (0.05–0.10)	742	
NR	0.06, 95%CI (0.04–0.11)	668	
**Ethnicity**			
Western	0.09, 95%CI (0.06–0.13)	694	0.171
Asian	0.06, 95%CI (0.04–0.09)	930	
**Study design**			
Retrospective	0.07, 95%CI (0.05–0.10)	1624	0.215
Prospective	0.06, 95%CI (0.03–0.12)	115	

## Discussion

This is the first study showing significantly lower discordance rates when using a strict method of HER2 positivity, entailing HER2 IHC scoring using the scoring system of Hoffman *et al*. with IHC 3+ defined HER2 positive or IHC2+ with additional positive ISH analyses. Applying the strict method of HER2 detection, 7% discordance between primary gastro-esophageal adenocarcinomas and corresponding metastasis was detected. When comparing discordance of strict vs. broader methods of HER2 detection, more discordance in regional metastases was observed when applying broader methods of HER2 assessment. This overall discordance effect is mainly due to more negative to positive conversions when using the broad(er) HER2 assessment methods compared to the strict method of HER2 assessment.

The use of a strict definition of HER2 positivity, has clear methodological advantages. As the use of the strict definition results in low discordance between primary tumor material and metastases, the question which tumor material to use to determine the HER2 status becomes less crucial. In the context of a clinical trial the use of a strict definition would enable the inclusion of a more uniform patient population, which would facilitate the interpretation of trial results. In terms of clinical benefit, it should be noted that in the ToGA trial, sub-group analyses of HER2 positive patients treated with trastuzumab in addition to standard chemotherapy regimens demonstrated survival benefit only for patients that were HER2 positive according to the strict definition of HER2 positivity^1^. Thus, adoption of the strict method of HER2 assessment could prevent overtreatment of patients. Nevertheless, recent retrospective analyses of trastuzumab treated HER2 ≤ IHC2+ advanced gastric adenocarcinomas demonstrated a HER2:CEP17 ratio of >3.69 and a HER2 gene copy number of >7.75 to be positive predictive factors of HER2 targeted therapy survival benefit, underscoring the need for further research into the question which patients derive benefit from trastuzumab treatment^35^.

Nevertheless, even if strict methods of HER2 positivity are applied, still 5% of the cases show negative to positive conversion and 18% show positive to negative conversion. This discrepancy between positive and negative conversion might be due to intra-tumor HER2 status heterogeneity and sampling errors possibly result in increased negative discordant cases. It has been shown that accurate HER2 testing of gastric carcinomas is optimal when at least 4–5 endoscopic biopsies are taken^36, 37^. Likewise the same might hold true for biopsies of distant metastatic sites, although in clinical practice this may be difficult to achieve.

The question is still open how this discordance influences response to treatment. Data on breast cancer patients indicate a reduced survival benefit of trastuzumab treatment in discordant HER2 status cases, irrespective of negative or positive conversion^38^. Whether the same holds true for gastro-esophageal adenocarcinomas needs to be elucidated. Therefore, for now, we advocate that patients with HER2 positivity according to the strict criteria in either primary tumor or metastasis should be offered HER2 targeted therapy. Given the fact that positive conversion occurs less frequently than negative conversion, we advocate the determination of the HER2 status, by means of the strict definition, on the primary tumor first (if available) and if negative, also on the metastasis. Unfortunately, little data is available on HER2 status discordance after neo-adjuvant therapy. Nowadays neo-adjuvant and adjuvant therapy is standard of care for many patients in the curative setting. More research needs to be conducted on the influence of (neo)adjuvant therapy on the discordance rate of HER2 status between primary tumors and corresponding metastases in gastro-esophageal adenocarcinomas.

## Conclusion

Treatment outcomes of different studies will be better comparable if selection of eligible patients for HER2 targeted therapy is based on the strict method of HER2 assessment. When using the strict method of HER2 assessment, 7% discordance is observed between primary gastro-esophageal adenocarcinomas and corresponding metastases. However, since 5% negative to positive conversions were observed, we advocate HER2 assessment first on the primary tumor and if negative also to test the corresponding metastasis.

## Methods

### Search strategy

The online search was performed on January 11^th^ 2016 in the PubMed, Embase and Cochrane library databases with no restriction for publication date. Medical subject headings (MESH) and text words for gastric and esophageal cancer where combined with those for HER2. Additionally, the reference lists of the included articles were manually screened to identify other relevant publications. The full search strategy is provided in the Supplementary Material (S1).

### Screening and selection of studies

Two reviewers (AC and LdW) independently screened the references by title and abstract, followed by full-text assessment. Any discrepancies were resolved by discussion until consensus was reached. In case of disagreement between the reviewers, a third reviewer (EtV) acted as in independent arbiter. Studies had to meet the following inclusion criteria: 1. the research population included patients with gastro- and/or esophageal cancer of any disease stage; 2. HER2 status of the tumors had to be determined using IHC and/or an ISH method on the primary tumor and at least one corresponding metastatic site; and 3. number of discordant cases to calculate a discordance rate should be available for extraction. Reviews, case reports, abstracts and phase I studies were excluded. Furthermore, articles without full-text in English were excluded. When articles had overlapping populations, the most recent publication was included. Endnote X7 was used to select and screen the literature.

### Data extraction and Outcomes

Data were extracted by two reviewers (LdW and EtV) according to a predefined protocol. All extracted data were double-checked and discrepancies were resolved by discussion with an independent arbiter (AC) until consensus was reached. The following study characteristics were extracted: last name of first author, year of publication and study design. Extracted population characteristics included: number of patients, ethnicity, location of primary tumor (esophageal/gastroesophageal junction (GEJ), gastric), (neo)adjuvant chemo(radio)therapy (previously received or not), type of metastasis (regional or distant metastasis), timing of metastasis (synchronous or metachronous), type of sampling of biopsy and metastasis (resection specimen or biopsy), method to detect HER2 status (IHC/ISH), scoring criteria (according to the Hoffman criteria^3^ or other) and number of discordant cases and sample sizes. All articles included were compliant to the Helsinki Declaration of 1975.

### Study quality assessment

Two reviewers (AC and EtV) independently evaluated the quality of all included studies. Discrepancies were resolved by discussion with an independent arbiter (LdW). Quality assessment was performed using an adapted version of the REporting recommendations for tumor MARKer prognostic studies (REMARK) criteria for biomarker studies^39^. For each of the seven selected quality criteria one point could be allocated to the article, in case of ambiguity half a point was allocated (Table 1). The sum of these points was used as a measure of the overall study quality. Studies were agreed to meet the quality criteria if at least 5 points were appointed to the study.

### Statistical analysis

Discordance rates between the HER2 status in the primary tumor vs. corresponding metastasis for each reported method of HER2 detection were calculated from the number of discordant cases and sample sizes as extracted from the articles, to calculate proportions and 95% Confidence Intervals (95%CI). The data were pooled with random-effect pair-wise meta-analysis to assess the differences in discordance rate between studies using both strict and broad(er) definitions of HER2 status. If multiple cohorts (e.g. with locoregional and with distant metastases) were evaluated in one study, both populations were included in the analyses if there were no overlapping patients. Pair-wise meta-analysis were performed in Review Manager V5. Random-effect single-arm meta-analyses were performed in R version 3.2.3. applying the strict method of HER2 assessment on the following subgroups: gastric, GEJ or esophageal cancer, the prevalence of negative conversion or positive conversion, type of metastasis (regional, distant and synchronous or metachronous). Sensitivity analyses were conducted on: received neoadjuvant therapy, study design, ethnicity and histology. Test heterogeneity was defined as p < 0.05 and I^2^ > 50%.

## Electronic supplementary material

Supplementary Information
