# Supplementary material for: Discordance in HER2 Status in Gastro-esophageal Adenocarcinomas: A Systematic Review and Meta-analysis
Source: Sci Rep. 2017 Jun 9;7:3135. doi: 10.1038/s41598-017-03304-9 (PMC5466678; doi:10.1038/s41598-017-03304-9)
Supplement: Supplementary file 1 — Supplementary Information [file 41598_2017_3304_MOESM1_ESM.pdf]

Discordance in HER2 Status in Gastro-esophageal Adenocarcinomas: A Systematic Review and Meta-analysis

Authors

A. Creemers*[1,2], E. ter Veer[2], L. de Waal[2], P. Lodder[3], G.K.J. Hooijer[4], N.C.T. van Grieken[5],M.F. Bijlsma[1], S.L. Meijer[4], M.G.H. van Oijen[2], H.W.M. van Laarhoven[2]

Affiliations

[1] Center for Experimental and Molecular Medicine (CEMM)/ Laboratory for Experimental Oncology and Radiobiology (LEXOR), AMC, Meibergdreef 9, 1105 AZ Amsterdam, The Netherlands

[2] Department of Medical Oncology, AMC, Meibergdreef 9, 1105 AZ Amsterdam, The Netherlands[3]

[3]Department of Methodology and Statistics/Department of Medical and Clinical Psychology, Tilburg University, Warandelaan 2, 5037 AB Tilburg, The Netherlands

[4] Department of Pathology, AMC, Meibergdreef 9, 1105 AZ Amsterdam, The Netherlands

[5]Department of Pathology, VUMC, De Boelenlaan 1117, 1081 HV Amsterdam, The Netherlands

Supplementary Information

S1

Database: **PubMed**
Date of search: 11 January 2016

#	Searches	Results
1	"Esophageal Neoplasms"[Mesh] OR "Stomach Neoplasms"[Mesh] OR "Carcinoma, Squamous Cell"[Mesh]	211578
2	(esophag*[tiab] OR oesophag*[tiab] OR gastric[tiab] OR gastroesophag*[tiab] OR gastrooesophag*[tiab] OR stomach[tiab] OR squamous[tiab] OR barrett*[tiab]) AND (cancer*[tiab] OR neoplas*[tiab] OR tumor*[tiab] OR tumour*[tiab] OR malignan*[tiab] OR carcino*[tiab] OR adeno*[tiab] OR metastas*[tiab])	224275
3	#1 OR #2	291861
4	"Receptor, ErbB-2"[Mesh] OR "Genes, erbB-2"[Mesh]	19505
5	HER2[tiab] OR HER 2[tiab] OR epidermal growth factor receptor 2[tiab] OR ERBB2[tiab] OR ERBB-2[tiab] OR ERB B 2[tiab] OR c-erbb-2[tiab] OR cerbb2[tiab] OR neu[tiab] OR neu protein[tiab] OR neu receptor[tiab] OR receptor neu[tiab] OR neuregulin receptor[tiab]	30150
6	#4 OR #5	32958
7	#3 AND #6	2464

Database(s): **Embase Classic+Embase** 1947 to 2016 January 08
Date of search: 11 January 2016

#	Searches	Results
1	exp *esophagus tumor/ or exp *stomach tumor/ or exp *squamous cell carcinoma/ or ((esophag* or oesophag* or gastric or gastroesophag* or gastrooesophag* or stomach or squamous or barrett*) adj3 (cancer* or neoplas* or tumor* or tumour* or malig* or carcino* or adeno* or metastas*)).ti,ab,kw.	294766
2	epidermal growth factor receptor 2/ or proto oncogene/ or (epidermal growth factor receptor 2 or HER2 or HER 2 or epidermal growth factor receptor 2 or ERBB2 or ERBB-2 or ERB B 2 or c-erbb-2 or cerbb2 or neu protein or	70928

	neu receptor or receptor neu or neuregulin receptor).ti,ab,kw.	
3	1 and 2	4222

Database: **Cochrane Central Register of Controlled Trials (Cochrane Library, January 2016)**
Date of search: 11 January 2016

#	Searches	Results
1	MeSH descriptor: [Esophageal Neoplasms] explode all trees	1059
2	MeSH descriptor: [Stomach Neoplasms] explode all trees	1779
3	MeSH descriptor: [Carcinoma, Squamous Cell] explode all trees	2192
4	(esophag* or oesophag* or gastric or gastroesophag* or gastrooesophag* or stomach or squamous or barrett*) near/3 (cancer* or neoplas* or tumor* or tumour* or malignan* or carcino* or adeno* or metastas*):ti,ab,kw (Word variations have been searched)	9589
5	#1 or #2 or #3 or #4	9603
6	MeSH descriptor: [Receptor, ErbB-2] explode all trees	474
7	MeSH descriptor: [Genes, erbB-2] explode all trees	50
8	epidermal growth factor receptor 2 or HER2 or HER 2 or epidermal growth factor receptor 2 or ERBB2 or ERBB-2 or ERB B 2 or c-erbb-2 or cerbb2 or neu protein or neu receptor or receptor neu or neuregulin receptor:ti,ab,kw (Word variations have been searched)	3575
9	#6 or #7 or #8	3575
10	#5 and #9, in Trials	143
